# Association between Genetic Polymorphism and Risk of von Willebrand Disease in Pakistan

**DOI:** 10.1155/2017/1070471

**Published:** 2017-12-20

**Authors:** Najma Arshad, Syed Kashif Nawaz, Riffat Iqbal, Muhammad Arshad, Farhana Musheer, Amber Naz, Iqra Mushtaq, Sara Jaleel

**Affiliations:** ^1^Department of Zoology, University of the Punjab, Lahore, Pakistan; ^2^Department of Zoology, University of Sargodha, Sargodha, Pakistan; ^3^Department of Zoology, Government College University, Lahore, Pakistan; ^4^University of Education, Lower Mall Campus, Lahore, Pakistan; ^5^Fatmeed Foundation, Lahore, Pakistan

## Abstract

von Willebrand disease (VWD) is an inherited, genetically and clinically heterogeneous hemorrhagic disorder. The most common cause of this disease is mutation in the gene that encodes protein von Willebrand factor (VWF) which is responsible for blood clotting. The current study was designed to investigate the role of genetic polymorphisms with the onset of VWD in population of Pakistan. Three exonic variants (c.3445T>C; c.4975C>T; c.7603C>T) from VWF gene were used for the genotyping purpose. The current study employed a case-control association design involving 43 VWD patients and 100 healthy controls from Pakistani population. The genetic reason of VWD was investigated using the allele specific PCR. The significant (*P* < 0.05) allelic association was found between all three exonic variants and VWD. The CT genotype of these variants was noticed to be associated with significantly higher risk of VWD [odds ratio (95% CI): 14.7 (4.546–47.98), 26.71 (7.281–97.98), and 21.5 (5.806–80.01) for c.3445T>C, c.4975C>T, and c.7603C>T, resp.] while genotypes CC (c.4975C>T) and TT (c.3445T>C and c.7603C>T) were having protective effect against the disease. However, replicated studies are needed for elaborating the role of these SNPs.

## 1. Introduction

von Willebrand disease (VWD) is a genetic problem that was discovered by Erik von Willebrand in 1926. Its symptoms include the bleeding disorder after minor injury or surgical treatment. Excessive bleeding after tooth extraction, gum bleeding, and nose bleeding are also included in its symptoms. The reason of VWD is the qualitative or quantitative variation in the von Willebrand factor (VWF). VWF is responsible for the platelet adhesion after damage of blood vessels. Endothelial cells and megakaryocytes are involed in synthesis of VWD. The locus for gene encoding VWF is at chromosome 12. The gene size is of 178 kb. A total of 52 exons are present in this gene. A partial pseudogene is present on chromosome 22 comprising exons 23–34. The mature VWF is made up of 2050 amino acids. VWF is helpful in the classification of VWD. Type 1 and Type 3 VWD are due to quantitative VWF defect. Type 2 VWD is due to qualitative defect of VWF [1, 2]. The prevalence of VWD is variable depending on the ethnicity and populations. In some areas, the patients with VWD are reported to be 1% of the population, while, in others, its prevalence is estimated to be 1 in 10,000 [3]. The most prevalent type of VWD was Type 1 with incidence of about 1 in 1000 (80% of all VWD types). This type occurs due to quantitative defect in VWF [4].

In Pakistan, the consanguineous marriage is preferred, due to which the population is at increased risk of inherited bleeding syndromes. Even autosomal recessive disorders have also been observed in different studies. According to Pakistan's demographic and health survey, more than sixty-six percent of marriages are consanguineous. In Karachi, the consanguineous marriages were about 58.7% in 2011. The prevalence of VWD in different regions of Pakistan has been summarised in Table 1.

The genetic reason of VWD is attributed to VWF. Mutations in gene for VWF affect the qualitative or quantitative variations leading to VWD. For local population of Pakistan, there exists least data showing the association between the genetic variations and VWD. The current study was conducted to find association of three candidate alleles with VWD. Allele specific PCR based strategy was used for the identification of alleles. The SNPs were selected due to their causative role reported in other populations. However, in Pakistani population there exists no data showing the role of these genetic variations with onset of VWD. HapMap-HCB and QC filters were used for selection of SNPs. After the use of Genome Variation Server 138 (http://gvs.gs.washington.edu/GVS138/), all the SNPs with monomorphic sites or deviated from HWE (*P* < 1 × 10^−4^) were excluded. Three SNPs [c.3445T>C, p.Cys1149Arg; c.4975C>T, p.Arg1659T^*∗*^; c.7603C>T, p.Arg2535T^*∗*^ (*∗* represents Ter)] were selected in VWF gene.

## 2. Material and Methods

### 2.1. Study Design

This was a case-control study. All procedures were in compliance with the Declaration of Helsinki. All subjects gave written consent before the beginning of the study, and the study protocol was approved by Board of Advanced Studies and Research (BASR). A brief questionnaire was designed for the information required according to the objective of experimental study. The questionnaire included the information regarding, age, blood group, gender, cousin marriage ratios in family, family bleeding history, and relation with any patients having bleeding disorder in family. Blood sampling was completed from August 2011 to August 2012. Blood samples of one hundred (100) normal healthy persons were obtained from general population of Lahore (Students of University of Punjab, New Campus) as control. Forty-three (43) VWD patients were contacted for the blood sampling. Blood taken in EDTA coated vials was stored at ultralow temperature, −30°C, till it was used for further analysis.

### 2.2. Comparison of Baseline Characteristics

The data of patients including age, symptoms of nose bleeding, gum bleeding, menorrhagia, and blood group was recorded as baseline characteristics and it was compared with control group using chi-square test.

### 2.3. Genetic Analysis

Genomic DNA isolation and detection were performed using the standard protocols [9]. Three SNPs (c.3445T>C; c.4975C>T; c.7603C>T) in VWF gene were used for the genotyping purpose. The sequences of primers and other relevant details for the genotyping are shown in Table 2. For the optimization of normal primers and mutation specific primers, the DNA of control (healthy control) and the DNA of patients of VWD were used as template. Reaction mixture was prepared as per instructions for DreamTaq Green PCR Master Mix (Thermo Fisher Scientific, USA). The optimization for PCR reaction was performed by adjusting the annealing temperature range from 50 to 60 degrees centigrade. Amplification of DNA was performed in a thermal cycler (BIOER TECHNOLOGY Co., Ltd., TC-XP-G, China). Program for PCR amplification was adjusted with initial denaturation at 94°C for five minutes followed by thirty-five cycles of denaturation at 94°C for 30 seconds, annealing at the different temperatures ranging from 50 to 60 degrees centigrade for 30 seconds, and extension at 72°C for 1 minute. On the completion of 35 cycles, final extension was performed at 72°C for 5 minutes. The results for PCR amplification were observed using 1% agarose gel under UV transilluminator. After the optimization of PCR conditions, the genotyping was completed for all the samples.

### 2.4. Statistical Analysis

The chi-square test was used for the analysis of Hardy-Weinberg equilibrium. Gene frequencies, allele frequencies, and differences in genotype and allele frequencies between different groups were also examined. Odds ratios were calculated to quantify the positive and negative association of genotype with disease. Chi-square test and other nonparametric tests were applied by SPSS® software, version 18 for Windows (SPSS Inc., Chicago, Illinois, USA), and MINITAB Student Version, release 12 for Windows (Minitab Inc.).

## 3. Results

The study was designed for the investigation of association between the genetic variation and the presence of VWD in local population of Pakistan.  Table 3 represents the baseline characteristics of the groups. The results show that both of the groups were significantly different on the basis of gender, nose bleeding, gum bleeding, menorrhagia, blood group B, and blood group O. There was no significant difference between the groups on the basis of age, blood group A, and blood group AB.

The genetic reason of VWD was investigated using the allele specific PCR. First of all, the PCR conditions were optimized for the genotyping on the basis of three variants (c.3445T>C; c.4975C>T; c.7603C>T). The experiments for optimization showed that annealing temperature for genotyping on the basis of c.3445T>C and for rs c.4975C>T was 60°C. The annealing temperature for the genotyping on the basis of c.7603C>T was 56°C. The optimized conditions were used for the genotyping of all the samples.

Table 4 represents the results of genetic analysis for the SNPs under observation. It shows that the allele frequency of local population for each SNP was deviant from HWE. Moreover, the results show strong association between the VWD and the studied genetic variants. Results for c.4975C>T variant indicate the protective effect of CC genotype (frequency was higher in control than patients). Based on odds ratio, CT genotype carriers were 14 times more prone to develop the VWD. In control group, no homozygous T genotype was found. Therefore, the result for association between VWD and this genotype could not be estimated. The analysis for T allele showed that its presence enhances the chances of disease. The variant c.3445T>C also showed the association with the VWD. TT genotype (c.3445T>C) was having protective effect against the occurrence of VWD. The TC genotype enhanced the chances of VWD 26-fold. No carrier of CC genotype was found in the control group. Therefore, the result for association between VWD and the CC genotype could not be estimated. The effect of C allele indicated its causative role for VWD. The SNP c.7603C>T was also found to influence the VWD. TT genotype was having protective effect, while TC genotype increased the chances of VWD 21-fold. CC genotype was absent in the control group. Therefore, the result for association between VWD and the CC genotype (c.7603C>T) could not be estimated. The presence of T allele increased the chances of VWD 42-fold.

Table 5 shows the haplotype frequencies and their association with VWD. The results show that haplotype TCT was present in both groups. Other haplotypes were restricted to a single group. CCT was present in the control group and was absent in VWD patients. The haplotypes ACT, ATT, and ATC were present in VWD patients and absent in control group. Due to absence of CC genotype in the control group, the association could not be estimated between that haplotype and VWD.

## 4. Discussion

von Willebrand factor (VWF) binds to the platelet and adheres to the site of injury. It also prevents the inactivation of circulating factor FVIII. The quantitative deficiency of VWF causes the Type 1 VWD, a compound heterozygous trait. In case of Type 2 VWD and Type 3 VWD, the inheritance pattern may be autosomal recessive [1, 2].

The present study focused on the association between the presence of VWD, a bleeding disorder, and the SNPs (c.3445T>C; c.4975C>T; c.7603C>T) in gene code for VWF using the DNA samples of VWD patients and healthy subjects. DNA was isolated by using kit method. Genotyping was performed using allele specific PCR.

The results for baseline characteristics of the samples indicate that the attribute of nose bleeding was present in diseased group with higher frequency (69.7%). In case of healthy group, only 7% of individuals showed these symptoms. Gum bleeding was also present in higher number of VWD patients (76.7%). Only 3% of healthy individuals showed the symptom of gum bleeding. Similar proportion was noticed for menorrhagia. In case of healthy group, 3% of individuals were showing the symptoms. The diseased group showed 55.8% prevalence of menorrhagia. The present observations show that the presence of nose bleeding, gum bleeding, and menorrhagia validates the symptoms of VWD.

A number of mutations were reported in VWF gene all over the world. These mutations may be point mutation, large deletions/duplications, missense mutation, and nonsense mutations. One missense mutation, responsible for the substitution of p.Tyr1584Cys, was found to be present in 8–20% cases of type 1 VWD patients in North America and Europe [10]. Other missense mutations, so far studied in association with VWD, cause the substitution of p.Arg845Gln in VWF [11]. The sequence encoded by the exon is helpful in proper attachment of VWF to the clotting factor. A variant c.7603C>T, in exon 28, causes the substitution of C with T resulting in the stop codon TGA rather than CGA [12]. The present observation indicates the strong association between the c.7603C>T and the occurrence of VWD. In current findings, the T allele frequency was higher in VWD patients. Only 2% of healthy individuals were having T allele. Similar findings, showing the presence of T allele in only patients, were reported by Zhang et al. [13].

A missense mutation (c.3445T>C) present in exon 26 is responsible for the accumulation of VWF in endoplasmic reticulum. It is also associated with the hindrance in the formation of VWF multimers [14]. The present finding indicates that c.3445T>C is strongly associated with the occurrence of VWD in local population of Pakistan. The C allele frequency was higher in patients and it increased the chances of occurrence of VWD. Eikenboom and coworkers [4] also observed the presence of this SNP in three unrelated patients. Nevertheless, the gap within 95% CI range is huge which is outcome of small sample size in the current study. For further clarity, the same study with large sample size is recommended.

A nonsense mutation in gene for VWF (c.7603C>T) was found to affect the binding of VWF to clotting factor FVIII [12]. In the present study, it was observed that this SNP (c.7603C>T) was strongly associated with the presence of VWD. The C allele frequency was higher in VWD patients. It indicates the role of C in affecting the function of VWF.

The haplotype based analysis for the study indicated that haplotypes were distributed on the basis of presence of disease. The only haplotype which was present in both of the groups was TCT. The haplotypes like CCT and CTC were present only in healthy group. Discordantly, the haplotypes like ACT, ATT, and ATC were present only in the VWD patients. The haplotype frequency for TCT was higher in both diseased and healthy individuals as compared to that of other haplotypes.

On the basis of the present findings, it can be concluded that the genetic variations like c.3445T>C, c.4975C>T, and c.7603C>T in gene code for VWF are important with reference to the onset of VWD in local population of Pakistan. The haplotype frequencies show that haplotypes like CCT and CTC are present in the normal healthy population. ACT and ATC haplotypes are present in VWD patients only. Only TCT is the haplotype which is present in both healthy and VWD patients. Clear picture of haplotype analysis could not be observed due to small sample size. The importance of the study can be described in terms of a large sample size for such a rare disease. The weakness of the study is the usage of only single method for the genotyping purpose. This study however provides a method based on allele specific PCR and also describes the importance of genetic variations like c.3445T>C, c.4975C>T, and c.7603C>T in gene-coding the VWF.

## Figures and Tables

**Table 1:** Prevalence of VWD in Pakistan.

Regions of Pakistan	Percentage of VWD cases among patients of bleeding disorder
Karachi	62.7% [5]
Khyber Pakhtunkhwa	12.74% [6]
Lahore	42% [7], 13% [8]
Rawalpindi	51% [7]

**Table 2:** Sequence and specification of primers.

Primer name	Primer sequence	Basic TM	Position	Length	Product
*Allele specific *					
*rs 61748511*					
Normal primer	5′ACTTGACAGGCAGGTGCACT3′	53.8°C	Reverse	20	213 bp
Mutated primer	5′ACTTGACAGGCAGGTGCACC3′	55.9°C	Reverse	20	
Counter primer	5′ATTGGTGACGCCCATAGTCC3′	53.8°C	Forward	20	
*Allele specific *					
*rs 61750595*					
Normal primer	5′AGGACTTTGAGACGCTCCCCC3′	58.3°C	Forward	21	459 bp
Mutated primer	5′AGGACTTTGAGACGCTCCCCT3′	56.3°C	Forward	21	
Counter primer	5′AAGGCAGAGGGTGGAATTGG3′	53.8°C	Reverse	20	
*Allele specific *					
*rs 61751296*					
Normal primer	5′AAAGACCTCCTCCTTCACTCC3′	54.4°C	Reverse	21	361 bp
Mutated primer	5′AAAGACCTCCTCCTTCACTCT3′	52.4°C	Reverse	21	
Counter primer	5′GCTCAGCTCAAACCAATGCT3′	53.0°C	Forward	20	

**Table 3:** Baseline characteristics.

Characteristics	Control	VWD	Total	*P*
	*N* = 100	*N* = 43	*N* = 143	
AGE, years (mean ± SEM)	33.06 ± 2.53	36.14 ± 5.24	34.8 ± 4.6	*P* = 0.557
Nose bleeding	07 (7%)	30 (69.7%)	37 (25.8%)	*P* < 0.001^*∗*^
Gum bleeding	03 (3%)	33 (76.7%)	36 (25.1%)	*P* < 0.001^*∗*^
Menorrhagia	03 (3%)	24 (55.8%)	27 (18.1%)	*P* < 0.001^*∗*^
Blood groups				
A	15 (15%)	06 (14%)	21 (15%)	*P* = 0.135
B	48 (48%)	16 (37%)	64 (45%)	*P* < 0.001^*∗*^
AB	12 (12%)	04 (09%)	16 (11%)	*P* = 0.139
O	15 (15%)	17 (40%)	32 (23%)	*P* < 0.001^*∗*^
Undiagnosed	10 (10%)	00	10 (06%)	*P* < 0.001^*∗*^

*t*-test was used for the comparison of diseased and control groups. *P* < 0.001^*∗*^ indicates the significant difference.

**Table 4:** Results for the genetic analysis on the basis of c.4975C>T, c.3445T>C, and c.7603C>T.

SNP	Genotypes/alleles	VWD	Control	Odds ratio (95% CI)	*P* value	HWE
		*N* = 42	*N* = 100			*χ* ^2^ (*P*)
c.4975C>T *N* (%)	CC	16 (38)	96 (96)	0.02 (0.007–0.832)	<0.0001^*∗*^	24.81^*∗*^ (0.000)
	CT	16 (38)	04 (04)	14.7 (4.546–47.98)		
	TT	10 (24)	00 (00)	Infinity (NaN-Infinity)^*ψ*^		
	C	48 (58.5)	196 (98)	Reference	<0.0001^*∗*^	
	T	34 (41.5)	04 (02)	34.7 (11.75–102.5)		

c.3445T>C *N* (%)	TT	08 (19)	97 (97)	0.00 (0.001–0.029)	<0.0001^*∗*^	33^*∗*^ (0.000)
	TC	19 (45)	03 (03)	26.71 (7.281–97.98)		
	CC	15 (36)	00 (00)	Infinity (NaN-Infinity)^*ψ*^		
	T	35 (41.6)	197 (98.5)	Reference	<0.0001^*∗*^	
	C	49 (58.4)	03 (01.5)	91.9 (27.14–311.3)		

c.7603C>T *N* (%)	TT	18 (43)	97 (97)	0.02 (0.00–0.08)	<0.0001^*∗*^	20.6^*∗*^ (0.000)
	TC	16 (38)	03 (03)	21.5 (5.806–80.01)		
	CC	8 (19)	00 (00)	Infinity (NaN-Infinity)^*ψ*^		
	T	51 (60.8)	197 (98.5)	Reference	<0.0001^*∗*^	
	C	33 (39.2)	03 (01.5)	42.5 (12.52–144.1)		

^*∗*^Significant association. ^*ψ*^Due to zero sample size in a group, the results show the failure in estimation and are mentioned here as “Infinity (NaN-Infinity).”

**Table 5:** Haplotype frequencies and their association with VWD.

Haplotypes	VWD	Control	OR (95% CI)
TCT	35	196	0.014 (0.005–0.004)
CCT	-	01	0 (0–NaN)^*ψ*^
CTC	-	03	0 (0–NaN)^*ψ*^
ACT	13	-	Infinity (NaN-Infinity)^*ψ*^
ATT	03	-	Infinity (NaN-Infinity)^*ψ*^
ATC	33	-	Infinity (NaN-Infinity)^*ψ*^

^*ψ*^Due to zero sample size in a group, the results show the failure in estimation and are mentioned here as “Infinity (NaN-Infinity).”

## References

[1] James P. D., Goodeve A. C. (2011). Von Willebrand disease. *Genetics in Medicine*.

[2] Taylor J. (2015). Von willebrand disease. *Hemostasis and Thrombosis*.

[3] Bloom A. L. (1991). Von Willebrand factor: Clinical features of inherited and acquired disorders. *Mayo Clinic Proceedings*.

[4] Eikenboom J. C. J., Hilbert L., Ribba A. S. (2009). Expression of 14 von Willebrand factor mutations identified in patients with type.1 von Willebrand disease from the MCMDM-1VWD study. *Journal of Thrombosis and Haemostasis*.

[5] Borhany M., Shamsi T., Naz A. (2011). Clinical features and types of von Willebrand disease in Karachi. *Clinical and Applied Thrombosis/Hemostasis*.

[6] Shahbaz N., Ayyub M., Ahmed S. (2008). Spectrum of von willebrand disease in Northern Pakistan. *Pakistan Journal of Pathology*.

[7] Khan M. K., Khan S. Q., Malik N. A. (2014). Spectrum of von Willebrand’s disease in punjab: clinical features and types. *Journal of Ayub Medical College*.

[8] Mohsin S., Aslam M., Hussain S., Suhail S. (2012). Clinical manifestations and complications of von Willebrand disease. *Journal of Rawalpindi Medical College*.

[9] Sambrook J., Fritsch E. R., Maniatis T. (1989). *Molecular cloning, a laboratory manual*.

[10] Lillicrap D. (2005). The molecular genetics of von Willebrand disease. *Haematologica Reports*.

[11] Mazurier C., Hilbert L. (2005). Type 2N Von Willebrand disease. *Current Haematology Reports*.

[12] Bowman M., Tuttle A., Notley C. (2013). The genetics of Canadian type 3 von Willebrand disease: Further evidence for co-dominant inheritance of mutant alleles. *Journal of Thrombosis and Haemostasis*.

[13] Zhang Z. P., Blombäck M., Nyman D., Anvret M. (1993). Mutations of von Willebrand factor gene in families with von Willebrand disease in the Åland Islands. *Proceedings of the National Acadamy of Sciences of the United States of America*.

[14] Denis C. V. (2002). Molecular and cellular biology of von willebrand factor. *International Journal of Hematology*.

